# Excellent mid-term osseointegration and implant survival using metaphyseal sleeves in revision total knee arthroplasty

**DOI:** 10.1007/s00167-020-05865-1

**Published:** 2020-01-31

**Authors:** Sebastian M. Klim, Florian Amerstorfer, Gerwin A. Bernhardt, Patrick Sadoghi, Georg Hauer, Lukas Leitner, Andreas Leithner, Mathias Glehr

**Affiliations:** Department for Orthopaedics and Trauma, Medical University of Graz, Auenbruggerplatz 5, 8036 Graz, Austria

**Keywords:** Revision total knee arthroplasty, Metaphyseal fixation, Sleeves, Osseointegration, Bone defect

## Abstract

**Purpose:**

Metaphyseal fixation in revision total knee arthroplasty (RTKA) is a very promising treatment option for extended bone defects. Currently published mid-term results remain limited. The purpose was to analyse the implant durability, the clinical and the radiological mid-term results in RTKA when using metaphyseal sleeves.

**Methods:**

Clinical and radiological follow-up examinations were performed in 92 patients (93 knees) with RTKA using hybrid fixation technique (cementless sleeves and stem). Radiographic measurements regarding osseointegration at the bone–sleeve interface were performed and the range of motion (ROM), a subjective satisfaction score (SSS), the American Knee Society Score (KSS), the Western Ontario and McMaster Universities Osteoarthritis Index (WOMAC) as well as the SF-36 Health survey were examined. Bone defects were analysed using the Anderson Orthopaedic Research Institute (AORI) classification.

**Results:**

No knee had to be revised due to aseptic loosening at the time of the follow-up (mean 6.3 years ± 2.3, minimum 2 years). Satisfactory radiographic osseointegration at the sleeve/bone interface was detected in 96.1% of cases. 17 knees (18.2%) had to be re-revised, 15 of them due to a recurrent infection and 2 due to aseptic reasons (mediolateral instability and a periprosthetic fracture). The median of the ROM (96°), SSS (8), KSS (87), WOMAC (9), SF-36 MCS (55) and SF-36 PCS (38) showed very satisfying results.

**Conclusion:**

No case of aseptic loosening was found in this large series of RTKA with extended bone defects using metaphyseal sleeve fixation. In this large retrospective series, it has been shown that this technique is an excellent treatment option for extended bone defects in RTKA surgery.

**Level of evidence:**

Retrospective cohort study, level III.

## Introduction

The rising number of revision total knee arthroplasty (RTKA) procedures performed [16] and the predicted ongoing trend [17] will increase the demand for a durable, versatile method of fixation. Patients benefit from an extended prosthesis lifetime and further revision surgery costs could be reduced. Bone defects are a major risk of failure in RTKA surgery and commonly categorized according to the Anderson Orthopaedic Research Institute (AORI) classification (type 1 to 3) [6]. Depending on the grading, there are various treatment options [4]. AORI type 1 defects are commonly treated with bone cement or bone allografts. For AORI type 2 treatment, metal augmentations are available in different sizes and forms (wedges, blocks). Technically demanding procedures using bone grafts (impaction grafting and structural allografts) are versatile methods used in extended AORI type 2b and type 3 bone defects [20].

Metaphyseal sleeves and cones are designed to meet the difficulties of compromised metaphyseal bone quality and can be used for AORI grade 2–3 defects [24]. In theory, an implant fixation using metaphyseal sleeves in combination with a stem allows the fixation of the prosthesis in two (meta- and diaphysis) of the three relevant zones, even if the epiphysis and parts of the metaphysis are severely compromised by defects [15]. This should significantly reduce aseptic loosening, which is seen as one of the most common causes (29.8%) for RTKA surgery [18]. Data on large mid-term cohorts are still rare in the literature, but the published short-term as well as the first mid-term studies on the use of metaphyseal sleeves in RTKA surgery report good results [3, 8, 14].

The purpose of this study was to survey the osseointegration at the bone–sleeve interface, implant survival rates as well as the clinical score outcome of porous coated metaphyseal sleeve fixation in RTKA. The hypothesis is that mid-term results of a large cohort will considerably strengthen the positive trend in the literature, and significantly improve the knowledge on the indication and application of this method of fixation in daily clinical practice.

## Patients and methods

**Fig. 1:**
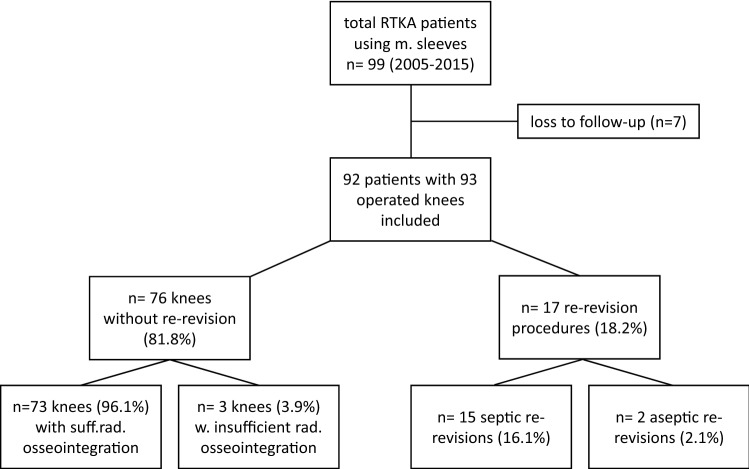
Flowchart depicting patient recruitment, outcome regarding re-revision rates, indications for re-revision and osseointegration

From 2005 to 2015, 99 patients (100 knees) underwent RTKA surgery using metaphyseal sleeves. Of these 99 patients, it was possible to perform clinical and radiological follow-up examinations in 92 patients (93%; 93 knees, Fig. 1). The remaining patients could not be re-evaluated for the following reasons: three died (of causes unrelated to the RTKA); four moved to a different country. Patients under custodianship and metaphyseal sleeves in combination with a hinged revision system or a megaprosthesis system were excluded from this study. In total 139 metaphyseal sleeves (91 tibial, 48 femoral) in combination with 172 stems (92 tibial, 80 femoral) were implanted. The indications for surgery were infections (52) and aseptic loosening (41). In 69 patients (74%), metaphyseal sleeves were implanted during the first revision and in 24 patients (26%) during the second or further revisions. Patient characteristics were as depicted in Table 1.

**Table 1:** Background characteristics of patients included in the study

Median of age at time of surgery (years)	68 (30–84)
Gender M:F (F%)	39:54 (58%)
Right knee:left knee (R%)	51:42 (55%)
Body mass index mean (kg/m^2^)	30 (17–41)
ASA score mean	2.8
ASA 1	1 (1%)
ASA 2	29 (31%)
ASA 3	51 (55%)
ASA 4	12 (13%)

*ASA *American Society of Anesthesiologists

In all patients, the metaphyseal sleeves were implanted in combination with the low contact stress (in combination with the varus valgus constraint insert) Complete Revision Knee System (DePuy Synthes, West Chester, Pennsylvania, USA) by a senior knee surgeon using the tibia first method with the balancing of the flexion gap. A broach in consecutive sizes was used to prepare the metaphyseal bone after reaming the intramedullary canal for stem insertion. This was done until a tight fit with rotational stability was achieved. In cases of extended AORI type 2b and 3 defects, the metaphysis was treated using bone grafts via the impaction grafting technique in addition to the metaphyseal sleeve fixation. For defect reconstruction in the epiphyseal area, metal augments (tibia and/or femoral) were used in combination with bone cement. Bone cement at the porous sleeve surface was strictly avoided to achieve sufficient osseointegration.

The established AORI classification was used to categorize metaphyseal bone defects on preoperative radiographs [6]. Pre-operative bone defects on the tibial and on the femoral sides were found as depicted in Table 2. Surgical complications were recorded using the classification of Goslings and Gouma which divides complications into six grades depending on the severity and possible necessity for a surgical re-intervention [7]. In six patients (6.5%), a “grade 1” surgical complication was detected (temporary disadvantage, no reoperation).

**Table 2:** Bone defect types in the tibia and femur treated with metaphyseal sleeves

AORI type	Tibia	Femur
2a	36 (39%)	13 (27%)
2b	47 (52%)	31 (65%)
3	8 (9%)	4 (8%)

*AORI* Anderson Orthopaedic Research Institute classification

The first follow-up (at 4.0 years ± 2.4) examination consisted of the American Knee Society Score (KSS), the Western Ontario and McMaster Universities Osteoarthritis Index (WOMAC), the SF-36 Health survey and a survey of stem tip pain [2, 10, 21]. The range of motion (ROM) was obtained preoperatively and at the time of follow-up using a goniometer. To further outline the patients’ subjective view regarding the condition of his knee, a subjective satisfaction score (SSS) was performed on a scale of 1 to 10, ranging from 1—‘extremely dissatisfied’ to 10—‘extremely satisfied’.

**Fig. 2:**
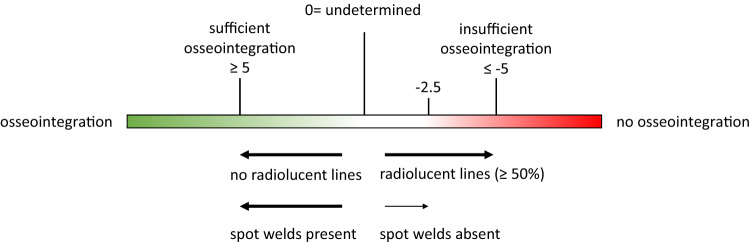
Radiographic classification of osseointegration according to Engh et al. [5]

A radiographic assessment (knee X-ray in anterior–posterior and lateral view, patella defilé, whole leg) was performed. The criteria described by Engh et al. were used to evaluate the level of osseointegration at the bone–sleeve interface [5]. Two blinded observers calculated the score twice within an interval of 6 weeks. A scale was used in which 5 points and more signifies a definitive radiological sign of osseointegration, 4 to − 4 points is a range of uncertainty (the higher the value, the better the chance for sufficient osseointegration). − 5 points and fewer are associated with no radiological signs of osseointegration. It was evaluated whether radiolucent lines (5 points if not present, − 5 points if extensively (50% or more) present) and spot welds were present (5 points if present, − 2.5 points if not present) on the bone-porous coated implant interface (Fig. 2) [5].

The second follow-up (at 6.3 years ± 2.3) consisted of a medical database search with a special focus on complications, re-revisions or other knee-related pathologies or hospitalisations of the included patients. Furthermore, the patients were contacted by telephone and asked about any pain, instability or other signs of septic/aseptic failure. The knee society score questionnaire was also conducted in this context by telephone. Additionally, the most current radiographs available (knee radiographs in the anterior–posterior and lateral view, taken during routine follow-up examinations) were assessed by the first author and compared to those of the first follow-up (at 4.0 years ± 2.4) in terms of newly occurring or progressive radiolucent lines, bone defects or other signs of aseptic loosening. This study was approved by the institutional review board of the Medical University of Graz (28-369 ex 15/16). Informed consent was obtained from all individual participants included in the study.

### Statistical methodology

Descriptive and explorative data analyses were performed using IBM SPSS Statistics (Version 25.0). We evaluated data with respect to parametric or non-parametric distribution using a Kolmogorov–Smirnov test where appropriate. To detect significant differences, we used the paired and unpaired *t* test. If parametric distribution was not given, the Mann–Whitney *U* test was performed. A *p* value of less than 0.05 was considered as statistically significant. For the radiographic evaluation of osseointegration, an inter- and intra-observer reliability analysis using Cohen’s kappa was performed to determine consistency among observers [13]. The following grading was used to rate the agreement: 0–0.2 (slight), 0.21–0.40 (fair), 0.41–0.60 (moderate), 0.61–0.80 (substantial) and 0.81–1.0 (almost perfect). With respect to sample size calculation, a post hoc power analysis according to Hoenig and Heisey was performed [9]. According to this method, post hoc power for differences with a *p* value < 0.05 reveals a value greater than 80% of power.

## Results

During the complete follow-up period (6.3 years ± 2.3), 17 knees (18.2%) had to be re-revised, 15 (16.1%) for septic reasons and 2 (2.1%) for aseptic reasons due to one knee with a mediolateral instability and one with a periprosthetic fracture (Fig. 1). No case of aseptic loosening or implant failure was found.

Successful radiographic osseointegration in 73 out of 76 patients without re-revision (96.1%) was detected. Insufficient radiographic signs of osseointegration were found in three patients (3.9%) at 4.0 years ± 2.4. The kappa of the inter- and intra-observer agreement rates for signs of osseointegration was 0.74 (substantial) and 0.85 (almost perfect), respectively (both *p* < 0.001). Patients with insufficient radiographic signs of osseointegration did not present any clinical signs of loosening in the follow-up examinations; therefore, no re-revision surgery was necessary at 6.3 (± 2.3) years. Moreover, no newly occurring or progressive radiolucent lines, bone defects or other signs of aseptic loosening were detected in the most recent radiographs at the second follow-up. In 17 (22.4%) patients, overall 37 bone resorption zones beneath the tibial tray were found. All these patients had signs of successful osseointegration at the bone sleeve interface (at 4.0 years ± 2.4).

Pre- and postoperative questionnaire results as depicted in Table 3. The mean ROM improved from 91° (range 40°–140°, preoperative) to 96° (range 25°–125°, postoperative) without a statistical significance. Leg alignment was analysed measuring the hip–knee–ankle axis. The normoalignment threshold was 3° for varus and valgus malalignments, respectively. Valgus malalignment in three patients and varus malalignment in six patients were detected (malalignment quota of 9.7%). Stem-tip pain was found tibially in three patients (3.2%) and femorally in none (at 4.0 years ± 2.4). According to the magnitude of differences between pre- and postoperative scores, we observed a post hoc power greater than 80% according to Hoenig and Heisey for the WOMAC, SSS, and SF-36 PCS with *p* values smaller than 0.05, and for inter- and intra-observer agreement rates with *p* values smaller than 0.01, each.

**Table 3:** Median of WOMAC, subjective satisfaction score, SF-36 MCS and PCS preoperatively and KSS at 4 (± 2.4) years and 6.3 ± 2.3 years, respectively, including *p* values

Score	Preoperative	At 4-year follow-up	*p* value
WOMAC	55 (± 8)	9 (± 14)	< 0.05
SSS	2 (± 1.2)	8 (± 2.4)	< 0.05
SF-36 MCS	64 (± 14)	55 (± 12)	n.s
SF-36 PCS	24 (± 8)	38 (± 9)	< 0.05
KSS	86 (± 20) at 4 years FU	87 (± 18) at 6.3 years FU	n.s

*KSS* American Knee Society Score, *WOMAC *Western Ontario and McMaster Universities Osteoarthritis Index, *SSS* Subjective Satisfaction Score, *SF-36 MCS* SF-36 Health Survey Mental Component Summary, *SF-36 PCS* SF-36 Health Survey Physical Component Summary

## Discussion

The main finding of this study was the excellent rate of sufficient osseointegration (96.1%) with no case that had to be re-revised due to aseptic loosening in 93 knees at a mean follow-up time of 6.3 years. However, the course of three cases with radiological signs of insufficient osseointegration (mean follow-up 6.0 years) remains to be seen in future follow-ups. The outcome of RTKA fixation using metaphyseal sleeves including aseptic and septic revision indications was examined. Data on larger mid-term cohorts remain rare in the literature, but the first results on the use of metaphyseal sleeves in RTKA surgery report excellent results (Table 4) [14, 22, 23].

**Table 4:** Previously published studies on the use of metaphyseal sleeves in RTKA (selection, since 2015)

Study	AORI bone defects	Number of knees treated	Number of sleeves	Mean follow-up (years)	Maximum follow-up (years)	Aseptic loosening
Graichen et al.	2 and 3 in tibia 2B and 3 in femur	121	119 tibial 74 femoral	3.6	6.1	4 (1.7%)
Martin-Hernandez et al.	1b and 2 in tibia 1b and 2 in femur	134	134 tibial 134 femoral	6	8.9	0 (0%)
Chalmers et al.	1–3 in tibia 1–3 in femur	227	144 tibial^a^ 249 femoral^a^	3.2	8	2 (0.8%)
Fedorka et al.	1–3 in tibia 1–3 in femur	50	49 tibial 30 femoral	4.9	7.8	2 (4%)
Wirries et al.	1–3 in tibia 1–3 in femur	47	44 tibial 30 femoral	5.0	6.9	3 (6.4%)
Watters et al.	2–3 in tibia 2–3 in femur	104	134 in total	5.3	9.6	0 (0%)
Present study	2–3 in tibia 2–3 in femur	93	91 tibial 48 femoral	6.3	12.3	0 (0%)

*AORI* Anderson Orthopaedic Research Institute

^a^68 femoral and 143 tibial sleeves cemented

The excellent clinical score results at follow-up are equivalent to previous study results [8, 14, 23]. The rate of stem-tip pain (tibial 3.2%—femoral 0%) is comparable to the findings of Graichen et al. (tibial 1.7%—femoral 1.4%) [8] and Martin-Hernandez et al. (tibial 2.2%) [14]. The six patients in which a “grade 1” surgical complication occurred included five confined, non-dislocated periprosthetic fractures and one iatrogenic vascular lesion. All complications were treated intraoperatively—the vascular lesion was sutured and four periprosthetic fractures (all four in the distal femur) were treated with cerclages. The other periprosthetic fractures were treated with postoperative weight-bearing restrictions and observation (non-weight-bearing for 6 weeks using crutches). All fractures healed clinically and radiographically. Barnett et al. (four cases) and Martin-Hernandez et al. (one case) described similar findings of bone resorption zones in the tibial tray area. However, successful osseointegration in the sleeve zones was confirmed in all patients [1, 14].

In the concept of zonal fixation, porous tantalum cones constitute a different approach to address the metaphysis. Mid-term studies regarding this technique show very encouraging results in terms of the aseptic loosening rate and osseointegration [11, 12, 19]. There are some differences between metaphyseal sleeves and cones that must be considered. While metaphyseal sleeves are a fixed part of the prothesis, cones can be used more flexibly as an independent part for the treatment of the bone defect. The connection to the prosthesis is made after the implantation. The potential drawback of this approach is the additional interface between cone and prosthesis. It remains to be seen if this constitutes a significant disadvantage regarding long-term fixation.

There are some limitations of this work: AORI defect types from grade 2 to 3 were included as well as revisions due to periprosthetic infection to represent the wide indication spectrum for metaphyseal sleeves. The drawback to this approach is a tolerable bias of results especially as metaphyseal sleeves are used in a variety of revision indications. Furthermore, no comparative cohort was included in the study. The subjective satisfaction data, WOMAC and SF-36 score were collected postoperatively for both the pre- and postoperative assessment which constitutes a limitation. The follow-up examination was not possible in four patients with a treatment-resistant prosthetic infection that led to amputation. It must be assumed that the scheduled examination would have negatively influenced the mean study outcome, due to chronic infection-related issues. Despite the large number of cases and the satisfying follow-up period, long-term results need to be awaited before making a final assessment on the use of metaphyseal sleeves in the treatment of extended bone defects in RTKA surgery.

The significant benefit as well as the clinical relevance of this piece of work must be underlined, as it represents one of the largest series of RTKA using sleeves with a mid-term follow-up period in the literature. By collecting the WOMAC and SF-36 as well as a subjective satisfaction score, a special focus was put on the postoperative satisfaction of patients. This will be even more necessary in future, as patients become more independent and have to be included in diagnostic and therapeutic decision-making.

## Conclusion

No case of aseptic loosening was found when using metaphyseal sleeves for implant fixation in RTKA patients. Metaphyseal sleeves show very good mid-term results regarding clinical scores and osseointegration. In this large retrospective series, it has been shown that this technique is an excellent treatment option for extended bone defects in RTKA surgery.

## References

[1] Barnett SL, Mayer RR, Gondusky JS, Choi L, Patel JJ, Gorab RS (2014). Use of stepped porous titanium metaphyseal sleeves for tibial defects in revision total knee arthroplasty: short term results. J Arthroplasty.

[2] Bellamy N, Buchanan WW, Goldsmith CH, Campbell J, Stitt LW (1988). Validation study of WOMAC: a health status instrument for measuring clinically important patient relevant outcomes to antirheumatic drug therapy in patients with osteoarthritis of the hip or knee. J Rheumatol.

[3] Bugler KE, Maheshwari R, Ahmed I, Brenkel IJ, Walmsley PJ (2015). Metaphyseal sleeves for revision total knee arthroplasty: good short-term outcomes. J Arthroplasty.

[4] Cuckler JM (2004). Bone loss in total knee arthroplasty: graft augment and options. J Arthroplasty.

[5] Engh CA, Massin P, Suthers KE (1990). Roentgenographic assessment of the biologic fixation of porous-surfaced femoral components. Clin Orthop Relat Res.

[6] Engh GA, Ammeen DJ (1999). Bone loss with revision total knee arthroplasty: defect classification and alternatives for reconstruction. Instr Course Lect.

[7] Goslings JC, Gouma DJ (2008). What is a surgical complication?. World J Surg.

[8] Graichen H, Scior W, Strauch M (2015). Direct, cementless, metaphyseal fixation in knee revision arthroplasty with sleeves-short-term results. J Arthroplasty.

[9] Hoenig JM, Heisey DM (2001). The abuse of power: the pervasive fallacy of power calculations in data analysis. Am Stat.

[10] Insall JN, Dorr LD, Scott RD, Scott WN (1989). Rationale of the Knee Society clinical rating system. Clin Orthop Relat Res.

[11] Kamath AF, Lewallen DG, Hanssen AD (2015). Porous tantalum metaphyseal cones for severe tibial bone loss in revision knee arthroplasty: a five to nine-year follow-up. J Bone Jt Surg Am.

[12] Lachiewicz PF, Watters TS (2014). Porous metal metaphyseal cones for severe bone loss: when only metal will do. Bone Jt J.

[13] Landis JR, Koch GG (1977). The measurement of observer agreement for categorical data. Biometrics.

[14] Martin-Hernandez C, Floria-Arnal LJ, Muniesa-Herrero MP, Espallargas-Donate T, Blanco-Llorca JA, Guillen-Soriano M (2017). Mid-term results for metaphyseal sleeves in revision knee surgery. Knee Surg Sports Traumatol Arthrosc.

[15] Morgan-Jones R, Oussedik SI, Graichen H, Haddad FS (2015). Zonal fixation in revision total knee arthroplasty. Bone Jt J.

[16] National Joint Registry for England W, Northern Ireland and the Isle of Man (2018) 15th Annual Report 2018, pp 124–147

[17] Patel A, Pavlou G, Mujica-Mota RE, Toms AD (2015). The epidemiology of revision total knee and hip arthroplasty in England and Wales: a comparative analysis with projections for the United States. A study using the National Joint Registry dataset. Bone Jt J.

[18] Sadoghi P, Liebensteiner M, Agreiter M, Leithner A, Bohler N, Labek G (2013). Revision surgery after total joint arthroplasty: a complication-based analysis using worldwide arthroplasty registers. J Arthroplasty.

[19] Schmitz HC, Klauser W, Citak M, Al-Khateeb H, Gehrke T, Kendoff D (2013). Three-year follow up utilizing tantal cones in revision total knee arthroplasty. J Arthroplasty.

[20] Sculco PK, Abdel MP, Hanssen AD, Lewallen DG (2016). The management of bone loss in revision total knee arthroplasty: rebuild, reinforce, and augment. Bone Jt J.

[21] Ware JE, Sherbourne CD (1992). The MOS 36-item short-form health survey (SF-36). I. Conceptual framework and item selection. Med Care.

[22] Watters TS, Martin JR, Levy DL, Yang CC, Kim RH, Dennis DA (2017). Porous-coated metaphyseal sleeves for severe femoral and tibial bone loss in revision TKA. J Arthroplasty.

[23] Wirries N, Winnecken HJ, Lewinski GV, Windhagen H, Skutek M (2019). Osteointegrative sleeves for metaphyseal defect augmentation in revision total knee arthroplasty: clinical and radiological 5-year follow-up. J Arthroplasty.

[24] Zanirato A, Formica M, Cavagnaro L, Divano S, Burastero G, Felli L (2019). Metaphyseal cones and sleeves in revision total knee arthroplasty: two sides of the same coin? Complications, clinical and radiological results-a systematic review of the literature. Musculoskelet Surg.

